# Bio-Based Wood Adhesives: Current Advances in Polymer Architecture and Structure–Property–Sustainability Integration

**DOI:** 10.3390/polym18141689

**Published:** 2026-07-09

**Authors:** Panya Dangwilailux, Natworapol Rachsiriwatcharabul, Putipong Lakachaiworakun, Visit Eakvanich, Wassachol Wattana, Wachara Kalasee

**Affiliations:** 1 Department of Engineering, King Mongkut’s Institute of Technology Ladkrabang, Chumphon Campus, Chumphon 86160, Thailand; panya.da@kmitl.ac.th (P.D.); visit.ea@kmitl.ac.th (V.E.); wassachol.wa@kmitl.ac.th (W.W.); 2 Department of Sustainable Industrial Management Engineering, Faculty of Engineering, Rajamangala University of Technology Phra Nakhon, Bangkok 10800, Thailand; natworapol.r@rmutp.ac.th (N.R.); putipong.l@rmutp.ac.th (P.L.)

**Keywords:** bio-based wood adhesives, wood-based panel, lignin and tannins, global warming potential, crosslinking chemistry

## Abstract

The development of bio-based adhesives has emerged as a viable strategy to reduce fossil-derived resin consumption in wood and wood-based panel applications. This review provides a polymer-focused assessment of adhesive systems derived from proteins, carbohydrates, lignin, and tannins, emphasizing molecular architecture, crosslinking chemistry, interfacial interactions, and structure–property relationships. Adhesive performance is primarily dictated by functional group density, crosslinking efficiency, and network topology. Protein-based adhesives rely on hydrogen bonding and covalent crosslinking with lignocellulosic substrates but require structural modification to improve hydrothermal stability. Carbohydrate-based systems, including starch and cellulose derivatives, offer reactive hydroxyl functionalities that enable oxidation, esterification, and etherification pathways for enhanced network formation. Lignin and tannins, characterized by phenolic and aromatic structures, facilitate condensation reactions and enable partial substitution of phenol in thermosetting resins, supporting low-formaldehyde or formaldehyde-free formulations. Hybrid polymer networks, particularly protein–carbohydrate and lignin-modified systems, demonstrate improved crosslink density, reduced hydrophilicity, and enhanced mechanical performance. Life cycle analyses indicate that increasing biogenic carbon content and minimizing fossil-based cross-linkers can lower global warming potential (GWP) and volatile organic compound (VOC) emissions. Overall, a structure–property–sustainability framework is proposed to guide molecular design and performance optimization of next-generation bio-based wood adhesives.

## 1. Introduction

Wood-based materials constitute a strategic class of renewable resources that support the construction, furniture, and engineered materials sectors while contributing to climate change mitigation through long-term carbon storage [1,2]. Their favorable strength-to-weight ratio, process-ability, and adaptability to composite design position wood and wood-based panels as key components of low-carbon material strategies [3]. The increasing use of bonded wood products—such as particleboard, fiberboard, and engineered wood composites—enhances resource efficiency, reduces production waste, and extends service life [4]. These characteristics align closely with international sustainability frameworks, including the Bio–Circular–Green (BCG) Economy and the United Nations Sustainable Development Goals (SDGs), particularly SDGs 9, 12, and 13 [5]. Within this material system, adhesives serve as a critical enabling technology that governs the mechanical performance, durability, and environmental footprint of wood-based products [6]. Historically, natural adhesives were employed for wood bonding as early as ancient Egyptian times [7]. However, the industrialization of wood-based panel manufacturing in the twentieth century led to the widespread adoption of synthetic, fossil-derived adhesives because of their high bonding strength, water resistance, and reliable processing under industrial conditions [4]. Despite these technical advantages, conventional synthetic adhesives are increasingly scrutinized due to their reliance on non-renewable resources, life-cycle greenhouse gas emissions, and concerns regarding toxic emissions and occupational health [8,9]. In response to tightening environmental regulations, climate policies, and net-zero commitments, bio-based adhesives derived from renewable resources have emerged as a strategic technological alternative [10,11,12]. From a policy perspective, the transition toward bio-based adhesive systems supports decarbonization pathways, reduces dependence on fossil resources, and advances circular economy objectives [13]. From a technological standpoint, research over recent decades has demonstrated the feasibility of producing adhesives from biomass-derived constituents—such as starches, proteins, lignin, and tannins—as partial or full substitutes for petrochemical resins [4,12]. Carbohydrates, including starch and related polysaccharides, have been extensively studied as renewable macromolecular platforms for adhesive development due to their abundant hydroxyl functionality and modifiable structure [14,15]. Accordingly, bio-based adhesive development is increasingly viewed not only as a materials innovation challenge but also as a system-level intervention linking biomass valorization, industrial sustainability, and climate policy implementation.

Thailand provides a relevant case study for integrating bio-based materials into national sustainability strategies. The country generates substantial quantities of agricultural residues, including pineapple leaf fiber, banana leaves, rice straw, sugarcane leaves, rice husk, oil palm empty fruit bunches, as well as palm fronds and shells [16]. These residues are largely underutilized and are often disposed of through open-field burning, resulting in severe air pollution and increased greenhouse gas emissions [17]. From both policy and technological perspectives, converting these residues into value-added wood-based panels offers a viable pathway to mitigate environmental impacts while strengthening domestic bio-based industries [2]. Agricultural residues are rich in cellulose, hemicellulose, and lignin, which are key structural biopolymers suitable for engineered wood products [10]. Their utilization can reduce reliance on plantation wood, enhance material circularity, and lower emissions along the production chain [3]. When combined with bio-based adhesive technologies, these approaches support integrated circular economy models and contribute to national and regional net-zero targets [13].

At the technological level, considerable attention has been devoted to bio-based adhesives derived from proteins, starches, and phenolic compounds. Protein-based systems, including those derived from cottonseed and soybean, exhibit favorable adhesive–wood interactions through hydrogen bonding and covalent linkages with cellulose and lignin [4,7]. However, limited water resistance remains a key technical challenge, typically addressed through chemical modification or crosslinking strategies [12,18]. Starch-based adhesives have similarly been improved via esterification and molecular structure modification to reduce hydrophilicity and enhance performance [15,19]. Lignin and tannins, particularly condensed tannins, represent another class of bio-based adhesive precursors with strong relevance to industrial deployment. Their aromatic structures facilitate condensation reactions and impart mechanical strength and thermal stability, making them attractive candidates for formaldehyde-free or low-emission adhesive formulations [4,9]. Nevertheless, challenges related to moisture resistance, long-term durability, and performance consistency at industrial scale continue to constrain widespread commercialization [11].

To ensure scientific consistency and methodological transparency within the context of the present review, the majority of the authors involved in this work systematically compiled, analyzed, and synthesized current knowledge related to bio-based wood adhesives. This review primarily focuses on adhesive systems derived from renewable biomass resources, including proteins, carbohydrates, lignin, and tannins, with particular emphasis on polymer architecture, crosslinking chemistry, interfacial interactions, and structure–property–sustainability relationships. The review further aims to critically evaluate recent advances in the molecular design and performance optimization of bio-based adhesive systems for wood and wood-based panel applications, particularly in relation to mechanical strength, hydrothermal stability, formaldehyde reduction, and environmental sustainability. To achieve these objectives, a comprehensive literature survey was conducted using internationally recognized scientific databases, including National Center for Biotechnology Information (NCBI)/PubMed, Scopus, ScienceDirect (Elsevier), Web of Science Core Collection, and Google Scholar. The literature search covered peer-reviewed research articles and review papers published between 2000 and 2025. Consistent keyword combinations and search criteria were systematically applied throughout the review process, including “bio-based wood adhesive”, “protein adhesive”, “soy protein adhesive”, “starch adhesive”, “carbohydrate-based adhesive”, “lignin adhesive”, “tannin adhesive”, “crosslinking”, “polymer network”, “formaldehyde-free adhesive”, “life cycle assessment”, “volatile organic compounds”, and “green adhesive”.

**Figure 1:**
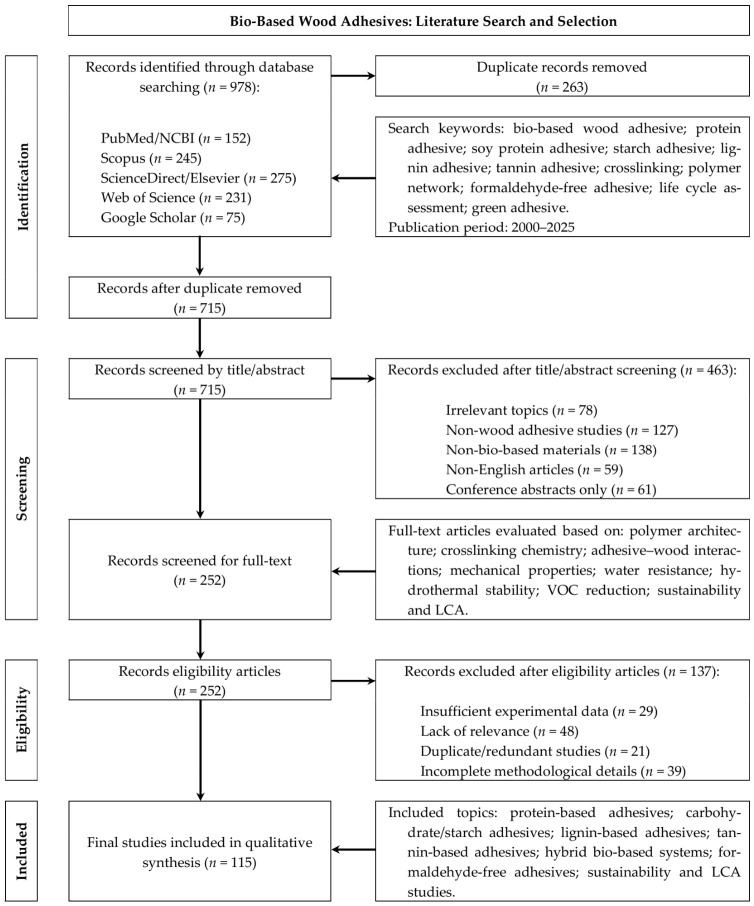
A PRISMA-type flow diagram for this review article.

The literature selection procedure was conducted in accordance with the Preferred Reporting Items for Systematic Reviews and Meta-Analyses (PRISMA) framework to ensure transparency, reproducibility, and minimization of duplicate records (Figure 1, Table S1). Only full-text articles published in English and indexed in internationally recognized scientific databases were considered. Particular emphasis was placed on studies directly addressing polymer network formation, curing mechanisms, adhesive–wood interfacial interactions, moisture resistance, mechanical performance, durability, and environmental assessment using life-cycle assessment (LCA) methodologies. Recent literature demonstrates a rapidly increasing global interest in bio-based adhesive technologies, particularly in the development of formaldehyde-free adhesive systems, hybrid and semi-interpenetrating polymer networks, and low-carbon materials for sustainable wood composite manufacturing. Increasing attention has also been directed toward reducing volatile organic compound (VOC) emissions and global warming potential (GWP) while simultaneously enhancing adhesive performance, durability, and industrial scalability. These developments strongly support broader international sustainability initiatives, including circular bioeconomy strategies and global net-zero emission targets.

Accordingly, current technology- and policy-driven research trends emphasize the development of hybrid bio-based adhesive systems, the integration of multiple biopolymers, and targeted chemical modifications to balance bio-based content, performance, and manufacturability [12,18].

This review critically examines the state of the art in bio-based adhesives for wood and wood-based panel applications from both technological and policy-oriented perspectives. It systematically addresses (i) the major classes of bio-based adhesive systems derived from proteins, carbohydrates, and phenolic compounds, and (ii) recent strategies for performance enhancement and industrial implementation, with particular emphasis on the utilization of agricultural residues and biomass resources within circular economy and net-zero frameworks. By integrating materials science, process technology, and sustainability considerations, this review aims to provide a structured reference for researchers, industry stakeholders, and policymakers seeking to accelerate the transition from fossil-based to bio-based adhesive systems in the wood and wood-based panel industry.

## 2. Methodology

This study employs a systematic literature review to collect, critically evaluate, and synthesize existing research on bio-based adhesives in the wood industry. The systematic approach was selected to ensure the accuracy, reliability, and completeness of the reviewed literature, while minimizing selection bias and enabling transparent and reproducible comparisons across studies. The review was conducted in two main stages: (i) identification and selection of relevant scholarly publications based on predefined search and screening criteria, and (ii) structured content mapping and analysis of the selected literature using predefined research questions. This framework enables the review to capture the current state of the art, identify research gaps, and outline emerging trends and future research directions. The methodology applied in each stage is described below.

### 2.1. Identification and Selection of Literature

A comprehensive search strategy was developed to address both primary and secondary topics related to bio-based adhesives in the wood industry. Boolean search strings were designed to cover key aspects of the field, including sources of bio-based adhesives, properties relevant to wood bonding performance, classification approaches, chemical structure and composition, and potential for industrial-scale application. Representative search terms included “bio-based adhesives” or “biomass-based adhesives,” “properties of bio-based adhesives for wood bonding,” “types or classification of bio-based adhesives,” “chemistry of bio-based adhesives,” and “future or potential of bio-based adhesives in the wood industry.”

The retrieved publications were screened according to predefined inclusion criteria, considering their relevance to the scope and objectives of the review, the depth and completeness of the scientific content, and the reliability of the source. Priority was given to peer-reviewed journal articles to ensure scientific rigor and data quality. This screening process resulted in a representative body of literature that reflects major developments, prevailing trends, and key challenges in research on bio-based adhesives for wood and wood-based products.

### 2.2. Content Mapping and Analysis

The selected studies were analyzed using a structured qualitative content-mapping approach based on a polymer-centered framework. The review was guided by predefined research questions and organized according to major natural polymer classes—proteins, lignin, carbohydrates, and tannins—with emphasis on molecular structure, adhesion mechanisms, modification strategies, and sustainability performance. The analysis addressed seven dimensions: (i) classification of bio-based adhesives by polymer source and hybrid systems; (ii) molecular architecture and reactive functional groups governing curing and network formation; (iii) structure–property relationships linking crosslink density to mechanical performance; (iv) interfacial adhesion mechanisms, including hydrogen bonding, covalent crosslinking, electrostatic interactions, and mechanical interlocking; (v) chemical and physicochemical modification strategies; (vi) durability-related properties, such as shear strength, moisture resistance, thermal stability, creep resistance, and viscoelastic behavior; and (vii) environmental indicators, including embodied carbon, biogenic carbon storage, global warming potential (GWP), and life-cycle assessment (LCA) metrics.

Data were systematically extracted and compared to identify consistent structure–property–performance relationships across polymer systems. Molecular-scale parameters, including functional group density and crosslinking reactions, were correlated with macroscopic adhesion performance in structural wood applications. Sustainability metrics—such as reductions in fossil resource use, VOC emissions, formaldehyde release, and greenhouse gas emissions—were evaluated alongside polymer design strategies. Quantitative LCA results, when available, were used to establish a direct relationship between molecular modification strategies and measurable reductions in global warming potential (GWP). This integrated framework provides a concise basis for assessing bio-based adhesives as high-performance, low-carbon materials within a circular bio-economy context.

## 3. Bio-Based Adhesives Derived from Natural Polymers

Bio-based adhesives represent a class of naturally derived polymeric materials that function as bonding agents. These materials are typically characterized by high molecular weight macromolecular structures, biocompatibility, and biodegradability, properties that have sustained interest in their development as alternatives to fossil-based synthetic adhesives [12,20]. The use of bio-based adhesives is widely recognized as an effective strategy to reduce dependence on petrochemical resources while mitigating environmental and human health impacts associated with volatile organic compound emissions and formaldehyde release [4,21]. Natural polymers are composed of fundamental molecular building blocks—such as monosaccharides in carbohydrates, amino acids in proteins, and phenylpropane units in lignin—interconnected into long-chain or three-dimensional macromolecular architectures [14,22]. These structural features result in large and complex polymer networks that exhibit physicochemical properties distinct from conventional synthetic polymers, including strong hydrogen bonding capability, polarity, and intrinsic hydrophilicity [23,24,25]. When applied to material surfaces, bio-based adhesives generate interfacial bonding forces that resist separation under mechanical loading, including tensile and shear stresses. Adhesion mechanisms typically involve hydrogen bonding, van der Waals interactions, covalent crosslinking, and mechanical interlocking with lignocellulosic substrates [4]. However, in their native form, most natural polymers exhibit inherent limitations, such as poor water resistance, limited thermal stability, and inferior bonding strength compared with synthetic thermosetting resins [20]. Consequently, research over the past decade has increasingly focused on enhancing the performance of bio-based adhesives through chemical and physical modification strategies. These strategies include functional group modification (e.g., oxidation, esterification, etherification), crosslinking reactions with multifunctional agents, and the development of hybrid systems that combine natural polymers with other bio-based or synthetic components to improve crosslink density and moisture resistance [12,21]. Such approaches aim to achieve performance metrics compatible with industrial wood panel manufacturing requirements.

Beyond their role as bonding agents, natural polymers can also function as matrices in composite materials. In composite systems, the matrix facilitates stress transfer between reinforcement fibers, maintains fiber alignment, protects fibers from environmental degradation, and suppresses crack propagation while providing cohesive interfacial bonding [26,27,28,29]. Within the broader context of natural polymer-based materials, the terms adhesive and matrix are therefore conceptually interconnected, as both describe polymer networks responsible for stress distribution and structural integrity. In this review, the terms are used in a consistent and complementary manner. Among sustainable natural polymer feedstocks with significant potential for adhesive and matrix applications are lignin, starch, proteins, and tannins. Each polymer class exhibits distinct chemical structures, adhesion mechanisms, and modification pathways. Lignin and tannins are rich in aromatic phenolic structures capable of condensation reactions; starch and other carbohydrates consist primarily of hydroxyl-functional polysaccharides; and proteins provide reactive amino, carboxyl, and amide groups that enable covalent and non-covalent interactions [4,14,22]. These structural differences result in variations in bonding performance, curing behavior, and durability. Accordingly, this review systematically examines each polymer category, emphasizing fundamental molecular structure, intrinsic performance limitations, and recent advances in modified and hybrid bio-based adhesive systems.

### 3.1. Proteins

Proteins are biopolymers composed of amino acids and constitute essential structural and functional components of living organisms [14,30,31,32]. Owing to the presence of diverse reactive functional groups—such as amino (–NH_2_), carboxyl (–COOH), and sulfhydryl (–SH) groups—proteins can interact effectively with wood substrates through hydrogen bonding, electrostatic interactions, and, in certain cases, covalent crosslinking [4,20]. These molecular characteristics confer significant potential for proteins as adhesive materials in wood products, and protein-based adhesives have been utilized historically for several centuries [4]. Despite their historical importance, the transition to modern industrial manufacturing systems has revealed several inherent limitations that restrict large-scale application. Protein-based adhesives generally exhibit high viscosity, short pot life, and pronounced sensitivity to moisture and water exposure [12]. Furthermore, variability in chemical composition due to biological origin, fluctuations in feedstock availability, and higher production costs compared with petrochemical resins present additional challenges to industrial competitiveness [20,21]. To overcome these limitations, contemporary research has increasingly focused on structural modification strategies aimed at improving water resistance, process-ability, and bonding strength. These strategies include molecular-level denaturation and unfolding treatments, chemical crosslinking using multifunctional agents, and the development of hybrid adhesive systems that integrate proteins with other bio-based polymers or synthetic resins [12,21]. Such approaches enhance crosslink density and network stability, enabling the rational design of environmentally sustainable adhesive systems that satisfy industrial performance requirements.

#### 3.1.1. Protein Chemistry

**Figure 2:**
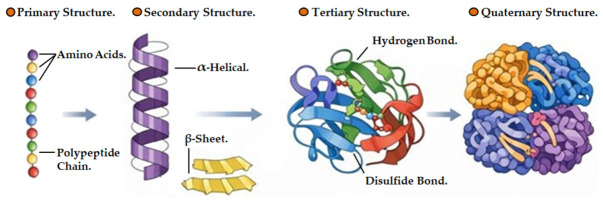
Hierarchical structural organization of proteins.

Proteins are high-molecular-weight macromolecules primarily composed of carbon, hydrogen, oxygen, nitrogen, and, in some cases, sulfur. Their fundamental building blocks are amino acids, of which approximately twenty standard types are arranged in diverse sequences, giving rise to a vast array of proteins with distinct physicochemical properties [14,30,31]. Adhesive performance is closely associated with the hierarchical structural organization of proteins, which comprises four levels: primary, secondary, tertiary, and quaternary structures in Figure 2. This multilevel architecture governs rheological behavior, interfacial adhesion mechanisms, and responsiveness to chemical modification, distinguishing protein-based adhesives from conventional synthetic polymer systems [4]. At the secondary structural level, proteins commonly adopt α-helical and β-sheet conformations stabilized by hydrogen bonding, whereas many thermoplastic synthetic adhesives exhibit crystalline or semi-crystalline domains [4,14,30]. The side chains of amino acids regulate hydrophilic–hydrophobic balance and provide reactive sites for crosslinking reactions and interactions with performance-enhancing additives [20]. Protein folding into tertiary and quaternary structures is governed by intra- and intermolecular interactions, including disulfide bond formation, ionic interactions, hydrophobic effects, and hydrogen bonding [14,32,33,34]. These structural features directly influence solubility, surface activity, curing behavior, and ultimate adhesion performance. Therefore, a comprehensive understanding of protein structure–property relationships is essential for the rational design of modified protein adhesives and protein-based hybrid systems with enhanced durability and mechanical performance in wood and wood-based panel applications.

#### 3.1.2. Plant Protein-Based Wood Adhesives

**Figure 3:**
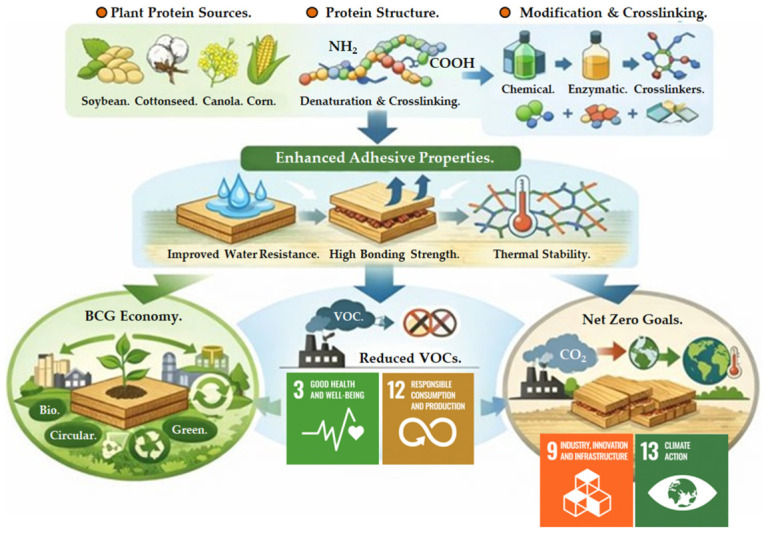
Schematically illustrates the structure–property–sustainability relationships of plant protein-based wood adhesives.

Plant proteins are renewable natural polymers and represent highly promising bio-based feedstocks for the development of environmentally friendly wood adhesives [35,36,37,38,39,40]. As summarized in Table 1, a wide range of plant protein sources—including soybean, cottonseed, canola, pea, and corn—have been extensively investigated for wood adhesive applications, either as primary raw materials or as by-products from agricultural, oilseed, and food processing industries [40,41,42,43,44]. The utilization of plant proteins as adhesive precursors directly aligns with the Bio–Circular–Green (BCG) Economy, as it promotes value-added use of biomass resources, enhances material circularity, and reduces dependence on petroleum-derived synthetic resins [45,46,47]. Plant proteins generally exhibit open molecular conformations and contain abundant functional groups, such as amino, carboxyl, and hydroxyl groups, which facilitate chemical modification and the formation of crosslinked networks [48,49]. These intrinsic structural characteristics enable effective tuning of rheological behavior, wood surface wettability, and interfacial adhesion strength, as comparatively summarized in Table 1. The mechanistic relationships among protein molecular structure, crosslinking reactions, and macroscopic adhesive performance are further illustrated in Figure 3 [50,51,52,53,54,55]. Importantly, replacing formaldehyde-based synthetic adhesives with plant protein-based systems significantly reduces emissions of hazardous volatile organic compounds (VOCs), thereby supporting SDG 3 and SDG 12 [56,57].

Among plant protein sources, soybean protein has been the most extensively studied due to its uniform composition, large availability, and low cost [36]. However, as shown in Table 1, native soybean protein adhesives suffer from inherently poor water resistance, which limits their commercial applicability, particularly for exterior wood products [35]. To overcome this limitation, numerous strategies have been proposed, including chemical and enzymatic modification, crosslinking reactions, and the incorporation of curing agents such as epoxy resins, melamine–formaldehyde, and polymeric methylene diphenyl diisocyanate (pMDI) [58,59]. These approaches substantially enhance water resistance, thermal stability, and bonding strength [60,61,62]. The effectiveness of these modification strategies, summarized in Table 1 and mechanistically depicted in Figure 3, demonstrates that increasing crosslink density is a key factor enabling bio-based adhesives to achieve performance levels comparable to petrochemical counterparts, while simultaneously reducing life-cycle carbon footprints and contributing to Net-Zero emission targets [56,57].

Beyond soybean protein, alternative plant proteins derived from cottonseed, camelina, pea, jatropha, and canola—many of which are industrial by-products—have attracted increasing attention [40,41]. Their utilization supports the Circular Economy by valorizing agricultural residues and minimizing waste generation. Property enhancement of these protein-based adhesives typically relies on hydrophobic modification and crosslinking strategies that improve water resistance and mechanical integrity of wood composites, thereby facilitating practical industrial adoption [56,59,60]. In addition, other plant proteins such as zein from corn and wheat gluten from cereal processing residues, despite limitations related to solubility or intrinsic protein properties, have demonstrated promising adhesive performance when appropriate dispersing agents and cross-linkers are employed [59,63,64,65,66]. The mechanistic framework presented in Figure 3 highlights how material design at the molecular level translates into improved adhesive performance and sustainability outcomes. This integrated approach supports SDG 9 and SDG 13 by fostering innovation in sustainable bio-based materials [56]. Overall, the comparative assessment in Table 1, together with the mechanistic overview in Figure 3, confirms that plant proteins constitute a highly versatile and sustainable platform for next-generation wood adhesives. These systems not only meet engineering performance requirements but also facilitate the transition toward low-carbon, resource-efficient material industries consistent with the principles of the BCG Economy, Net-Zero emissions, and the United Nations Sustainable Development Goals (SDGs).

#### 3.1.3. Protein–Carbohydrate-Based Bio-Wood Adhesives

**Figure 4:**
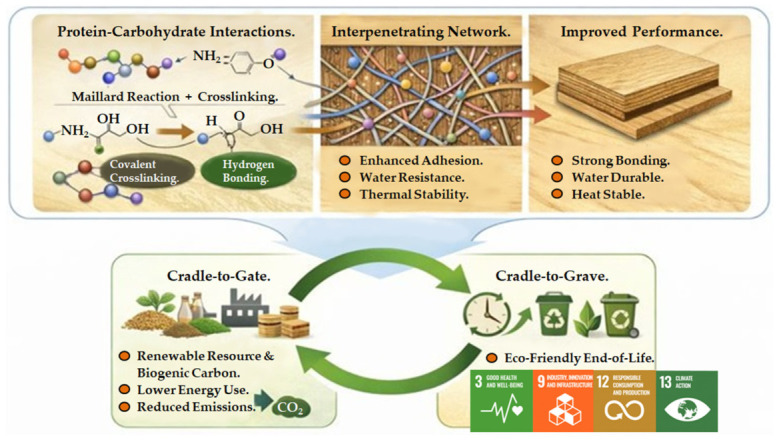
Mechanics and graphical summary of protein–carbohydrate-based bio-wood adhesives.

The incorporation of carbohydrates as functional additives in plant protein-based wood adhesive formulations represents a highly promising strategy for the development of environmentally benign bio-adhesives with performance suitable for industrial applications [67,68,69]. This approach is intrinsically aligned with the principles of the Bio–Circular–Green (BCG) Economy, as it promotes value-added utilization of renewable biomass resources, enhances circularity through the valorization of agricultural and agro-industrial by-products, and reduces reliance on petroleum-derived synthetic resins [18,40,68,69]. From a life cycle assessment (LCA) perspective, replacing conventional synthetic adhesives with systems composed of plant-derived proteins and carbohydrates has the potential to mitigate environmental impacts across multiple life-cycle stages, particularly during raw material acquisition and manufacturing [18,70,71]. These bio-based feedstocks typically require lower processing energy and inherently contain biogenic carbon, thereby contributing to a reduced overall carbon footprint of both adhesive formulations and wood-based composite products compared with petrochemical counterparts [40,68]. Previous studies have demonstrated that soy protein-based adhesives can achieve significant improvements in bonding strength and water resistance through the incorporation of glucose or sucrose at optimized ratios [36,72,73,74,75]. As schematically illustrated in Figure 4, the primary mechanism underlying these enhancements is the Maillard reaction between amino groups in protein chains and carbonyl groups in carbohydrates [39,76,77,78]. This reaction leads to increased crosslink density, reduced molecular mobility, and decreased hydrophilicity of the adhesive network. Consequently, plywood and wood composites bonded with such formulations exhibit markedly improved water durability and mechanical performance [77,78]. From an LCA standpoint, enhanced durability also contributes to impact reduction during the use phase, as prolonged service life lowers the frequency of repair or replacement, thereby reducing cumulative greenhouse gas (GHG) emissions over the product lifetime [77]. In addition to low-molecular-weight sugars, the combined use of structural carbohydrates—such as starch, cellulose, and xylan—with soy protein or cottonseed protein has been reported to yield mechanical performance comparable to other advanced modified protein-based adhesive systems [79,80]. As conceptually summarized in Figure 4, blending water-soluble polysaccharides with plant proteins facilitates the formation of an interpenetrating and synergistic network between the adhesive matrix and wood cell wall components [80]. This interaction improves adhesive penetration into wood substrates due to reduced viscosity, while simultaneously enhancing water resistance and thermal stability through hydrogen bonding and physical entanglement [77,80]. From an energy perspective, lower viscosity enables processing at reduced temperature and pressure, potentially decreasing manufacturing energy demand and associated process-level carbon emissions [56].

The adhesion mechanism of protein–carbohydrate-based adhesives is inherently multiscale and multifunctional, involving a combination of chemical bonding (covalent crosslinking and hydrogen bonding) and mechanical interlocking at the wood–adhesive interface [40,56,57]. As highlighted in Figure 4, these molecular- and microstructural-level interactions translate directly into macroscopic improvements in adhesive strength, durability, and environmental performance. Beyond enhanced bonding performance, such bio-based adhesive systems substantially reduce emissions of hazardous volatile organic compounds (VOCs) during both the use phase and at the end-of-life (EoL) stage [81,82,83]. Consequently, they provide clear benefits in terms of human health protection and environmental impact mitigation, while supporting Net-Zero emission targets and contributing to SDG 9 and SDG 12 [56].

To systematically evaluate the environmental sustainability of protein–carbohydrate-based wood adhesives, future studies should adopt comprehensive LCA approaches encompassing both cradle-to-gate and cradle-to-grave system boundaries, as conceptually integrated into the graphical framework shown in Figure 4. Within a cradle-to-gate framework, the assessment should include biomass cultivation, harvesting, and the utilization of agricultural and food-processing by-products, as well as protein and carbohydrate extraction, conditioning, and adhesive manufacturing up to the factory gate. Compared with petrochemical adhesives, bio-based systems are expected to exhibit lower environmental burdens at this stage due to the use of renewable feedstocks, reduced cumulative energy demand, and the sequestration of biogenic carbon in protein and polysaccharide structures, resulting in lower GHG emissions per functional unit.

Moreover, adhesive formulations relying on protein–carbohydrate interactions can reduce or eliminate the need for high-carbon-intensity synthetic cross-linkers, thereby lowering climate change impacts as well as human and eco-toxicity potentials during production. Nevertheless, cradle-to-gate assessments should also account for water and energy consumption associated with washing or pretreatment steps to ensure a balanced and transparent environmental evaluation. Under a cradle-to-grave framework, the assessment extends to the use phase and end-of-life of wood products bonded with bio-based adhesives. Improved water resistance and thermal stability—arising from Maillard reactions and interpenetrating network structures depicted in Figure 4—play a critical role in extending product service life. This enhanced durability reduces replacement frequency and contributes to a substantial decrease in cumulative life-cycle GHG emissions.

At the end-of-life stage, biomass-derived adhesives exhibit greater potential for environmentally favorable management options, including recycling, energy recovery as biofuel, or biodegradation under appropriate conditions. These pathways contrast sharply with conventional synthetic adhesives, which typically possess highly stable molecular structures that limit post-use treatment and contribute to long-term environmental burdens. Overall, cradle-to-gate and cradle-to-grave LCA frameworks consistently indicate that protein–carbohydrate-based bio-wood adhesives offer significant potential for reducing carbon footprints at both the manufacturing and full product life-cycle levels. This material design strategy supports the BCG Economy, advances progress toward Net-Zero goals, and contributes meaningfully to SDG 9 and SDG 12.

#### 3.1.4. Factors Influencing the Performance and Environmental Impacts of Plant Protein-Based Wood Adhesives

The performance of plant protein-based wood adhesives is governed by multiple interrelated factors, particularly the presence, composition, and physicochemical characteristics of carbohydrates within the adhesive matrix. Several studies have reported that water-soluble carbohydrates and low-molecular-weight protein fractions are closely associated with reduced wet bonding strength due to their hydrophilic nature and limited network-forming capability [84,85,86]. Both the total carbohydrate content [84] and carbohydrate type [19] exert a direct influence on water resistance and bonding performance. These properties subsequently affect life cycle assessment (LCA) outcomes, especially in relation to product durability, service life, and replacement frequency.

The removal of water-soluble carbohydrates from protein-rich raw materials—such as soybean meal and cottonseed meal—has been widely demonstrated as an effective strategy to reduce adhesive hydrophilicity and enhance water resistance [80,87,88]. For instance, aqueous washing treatment increased the protein content of soybean meal from 49.6% to 66.2%, resulting in significant improvements in both dry and wet shear strength of bonded wood composites [19]. Similar improvements have been reported for washed cottonseed meal adhesives, where enhanced crosslinking density and reduced carbohydrate interference contributed to improved moisture durability [73]. Although washing and purification processes require additional water and energy inputs, LCA-based analyses indicate that improvements in bonding durability and extended service life can compensate for these upstream impacts [12,75]. In many cases, longer-lasting adhesive performance reduces the frequency of panel replacement and maintenance, thereby lowering cumulative greenhouse gas emissions over the product life cycle. Moreover, the selection of functional additives and chemical modification strategies plays a decisive role in optimizing both mechanical performance and environmental outcomes. Crosslinking approaches involving epoxy compounds, polyamidoamine–epichlorohydrin (PAE), glyoxal, tannins, and lignin derivatives have been extensively investigated to enhance water resistance and cohesive strength [4,7,10,80]. Protein extraction and denaturation methods, including alkaline treatment, enzymatic hydrolysis, and thermal unfolding, also significantly affect adhesive reactivity and environmental footprint [32,89,90]. Process designs that minimize hazardous chemical usage, reduce energy-intensive operations, and prioritize agricultural by-products as feedstocks are therefore essential to lowering carbon footprints and supporting the sustainable advancement of bio-wood adhesive industries under the Bio–Circular–Green (BCG) Economy framework. Such integrated strategies directly contribute to long-term climate mitigation efforts, including alignment with SDG 13.

#### 3.1.5. Animal Protein-Based Wood Adhesives: Performance Development and Sustainability Perspectives

Animal-derived proteins, particularly collagen and gelatin obtained from bones and hides, as well as blood proteins recovered from slaughterhouse by-products, represent well-established raw materials for bio-based wood adhesives. Historically, these adhesives have been widely used in woodworking, furniture manufacturing, and the conservation of cultural heritage artifacts, reflecting their compatibility with lignocellulosic substrates and long-term durability. Typically, animal protein adhesives are solid at ambient temperature and are dissolved or dispersed in water prior to application to adjust the solid content and viscosity. Mild heating facilitates flow-ability and promotes the formation of a three-dimensional polymeric network through hydrogen bonding, electrostatic interactions, and intermolecular chain entanglement during curing and drying.

Recent research has focused on overcoming the inherent limitations of animal protein adhesives, particularly their sensitivity to moisture. For example, modifications using bio-based cross-linkers, such as tannins and lignin derivatives, have been reported to significantly enhance mechanical strength and water resistance [77,78,91]. Enzymatic treatments, especially with transglutaminase, have also been shown to improve cohesive strength and network stability in gelatin- and collagen-based adhesive systems [7,77]. In addition, blood protein-based adhesives have been developed for plywood and engineered wood applications, where chemical modification and blending strategies effectively improved bonding performance under humid and elevated-temperature conditions [79,80,81,82]. These studies demonstrate that traditional animal protein adhesives can be technologically upgraded to meet contemporary industrial standards. From a sustainability perspective, animal protein-based adhesives are closely aligned with the Bio–Circular–Green (BCG) Economy framework, as they valorize by-products from the livestock and meat-processing industries, thereby reducing waste and increasing resource efficiency. The conversion of residual biomaterials into value-added adhesive products promotes circular material flows and decreases reliance on petroleum-derived synthetic resins. Life-cycle assessments reported in recent studies indicate that substituting fossil-based adhesives with bio-based alternatives can reduce greenhouse gas emissions and overall environmental impact [4,12,71]. Consequently, the development of animal protein-based adhesives contributes to Net-Zero emission strategies and supports global sustainability agendas, particularly SDG 9, SDG 12, and SDG 13, through the advancement of sustainable industrial innovation and responsible production systems.

### 3.2. Lignin

Lignin is the second most abundant renewable biopolymer after cellulose, accounting for approximately 20–28% of the volume of wood [22,92]. Rather than being a single, well-defined substance, lignin comprises a heterogeneous group of complex aromatic polymers with structural variability depending on plant species and extraction processes [93]. Industrial isolation methods, such as kraft, sulfite, and organosolv pulping, significantly influence the chemical structure, molecular weight distribution, and reactivity of the resulting lignin fractions.

In native wood, lignin functions as a natural binder that cements cellulose fibers together. While cellulose provides the primary mechanical strength, lignin enhances structural rigidity and contributes to the superior strength-to-weight ratio of wood [22]. Moreover, lignin exhibits partial thermoplastic behavior, meaning that it can soften and flow under elevated temperature or combined heat and moisture conditions. This property is fundamental to the production of high-density fiberboard and hardboard, where heat and pressure promote fiber–fiber contact and self-bonding without the need for additional synthetic adhesives [94,95,96]. Despite these advantageous characteristics, the direct utilization of lignin as a wood adhesive remains challenging. Major limitations include its relatively high molecular weight, structural heterogeneity, limited number of reactive functional groups, and the technical complexity associated with its separation and purification from lignocellulosic biomass [92,93,97]. These factors often result in lower reactivity and bonding performance compared with conventional petroleum-derived resins. Nevertheless, the high availability and low cost of lignin—particularly as a by-product of the kraft pulping industry—have stimulated extensive research into its chemical modification and integration into bio-based adhesive systems. Strategies include partial substitution of phenol in phenol–formaldehyde resins, the development of lignin–formaldehyde adhesives, and the synthesis of lignin-based epoxy and polyurethane systems [4,12,98,99,100]. Collectively, these studies highlight the significant potential of lignin as a key feedstock for the development of sustainable wood adhesives and for reducing dependence on fossil-based raw materials.

#### 3.2.1. Chemistry of Lignin

Lignin is a complex aromatic biopolymer composed of phenolic structural units formed via radical polymerization of three primary monolignol precursors: coniferyl alcohol, sinapyl alcohol, and p-coumaryl alcohol [101,102,103]. These monomers originate from the plant phenylpropanoid biosynthetic pathway and serve as the fundamental building blocks of lignin.

**Figure 5:**
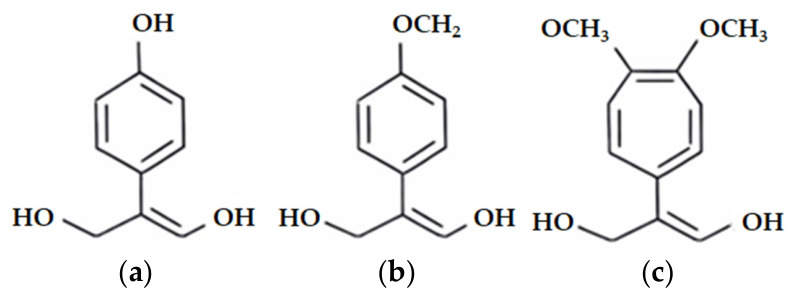
Chemical structures of the three primary monolignols of lignin: (**a**) p-coumaryl alcohol (H); (**b**) coniferyl alcohol (G); (**c**) sinapyl alcohol (S). Differences in methoxy substitution determine the formation of H, G, and S units and influence ortho reactivity, condensation degree, and crosslinking behavior in adhesive systems.

The chemical structures of the monolignol units and their methoxy substitution patterns are illustrated in Figure 5. Lignin consists of three principal phenylpropane units: p-hydroxyphenyl (H), guaiacyl (G), and syringyl (S). The H unit contains no methoxy groups, the G unit contains one methoxy group, and the S unit contains two methoxy groups on the aromatic ring [22,102,103,104]. These structural differences directly influence the number of reactive ortho positions available for electrophilic substitution and determine the degree of condensation within the lignin macromolecule. The monolignol units are interconnected through a variety of linkages, among which the β–O–4 ether bond is the most abundant in native lignin. In addition, condensed carbon–carbon linkages, such as β–β, β–5, and 5–5 bonds, contribute to the formation of a highly irregular and three-dimensional network structure. Owing to this diversity of interunit linkages and random coupling reactions, lignin does not possess a uniform repeating structure typical of synthetic polymers [101,103,104,105]. The relative abundance of H, G, and S units varies depending on biomass source and extraction process [92]. Softwood lignin is predominantly composed of G units, hardwood lignin contains both G and S units, whereas agricultural residues generally exhibit a higher proportion of H units. These compositional differences significantly affect lignin reactivity, molecular weight distribution, condensation degree, and suitability for adhesive applications. During industrial pulping processes, such as kraft or sulfite pulping, partial cleavage of β–O–4 linkages and secondary condensation reactions occur, leading to structural heterogeneity and a broad molecular weight distribution in technical lignins [93,101,103]. Consequently, applied research typically characterizes lignin in terms of average physicochemical parameters—such as molecular weight, phenolic hydroxyl content, and overall reactivity—rather than by a precise structural formula.

The chemical characteristics depicted in Figure 5 are particularly relevant to adhesive design. The availability of free ortho positions in H and G units governs the reactivity of lignin toward electrophilic crosslinking agents, including formaldehyde or bio-based alternatives [4,101,103]. Therefore, a comprehensive understanding of lignin monomer structure provides a critical foundation for developing bio-based resins with optimized mechanical performance and water resistance for wood composite applications.

#### 3.2.2. Lignin-Based Phenol–Formaldehyde Wood Adhesives

In phenol–formaldehyde (PF) resin synthesis, formaldehyde undergoes electrophilic substitution at the ortho or para positions of phenolic rings to form methylol phenol intermediates, which subsequently condense into a crosslinked three-dimensional polymeric network [101,102,103].

The ability of lignin to partially substitute phenol depends strongly on its structural unit composition. In S units, both ortho positions are blocked by methoxy substituents, thereby limiting reactivity toward formaldehyde. In contrast, G units possess one free ortho position, while H units contain two free ortho positions, resulting in higher chemical reactivity [105,106]. Accordingly, softwood-derived lignins, which are rich in G units, are generally more suitable for partial phenol substitution in PF resin formulations than hardwood lignins [101,105]. Numerous studies have demonstrated that kraft lignin can replace a substantial proportion of phenol without significantly compromising the mechanical properties of wood composites. In certain formulations, lignosulfonates have been reported to substitute phenol at levels approaching 50% wt [4,101].

Replacing fossil-derived phenol with lignin contributes to reduced petroleum consumption and lower greenhouse gas emissions along the production chain. This strategy also mitigates long-term raw material price volatility and aligns with bio-circular economic models that emphasize efficient utilization of renewable biomass resources.

#### 3.2.3. Formaldehyde-Free Lignin-Based Wood Adhesives

Formaldehyde is classified as a human carcinogen, and regulatory restrictions on formaldehyde emissions are becoming increasingly stringent worldwide. Consequently, significant research efforts have focused on developing formaldehyde-free wood adhesives in which lignin serves as the primary structural polymer component [101,102,106].

A representative example involves adhesive systems based on kraft lignin and polyethyleneimine (PEI), where crosslinking occurs through reactions between lignin hydroxyl groups and amine functionalities of PEI without the need for formaldehyde [107,108]. Patel et al., 2025 [83] reported that a lignin-to-PEI ratio of 2:1 yielded satisfactory shear strength and water resistance suitable for plywood applications, demonstrating the feasibility of lignin-centered bio-adhesive systems. Formaldehyde-free lignin adhesives offer multiple advantages, including reduced indoor volatile organic compound emissions, improved occupational health and safety, and decreased reliance on petrochemical feedstocks [109]. From a life cycle assessment perspective, such bio-based adhesives exhibit strong potential to lower carbon footprints, support net-zero emission targets, and promote sustainable development within the wood industry through the integration of renewable biomass resources.

### 3.3. Carbohydrate

#### 3.3.1. Molecular Architecture and Reactive Functionalities of Carbohydrates

Carbohydrates are bio-based polymers composed of monosaccharide units interconnected through glycosidic linkages, with an empirical formula of (CH_2_O)_n_. Their molecular architecture is characterized by multiple hydroxyl (–OH) groups located at the C2, C3, and C6 positions of the pyranose ring, which play a critical role in intermolecular interactions and chemical reactivity [14]. The high density of hydroxyl functionalities enables extensive hydrogen bonding, thereby governing the physicochemical and adhesive properties of carbohydrate-based systems [110,111].

In cellulose, β-D-glucopyranose units are linked via β(1 → 4) glycosidic bonds, resulting in a linear polymer chain capable of antiparallel packing into crystalline domains known as cellulose I [110,112]. This crystalline arrangement is stabilized by a dense network of intramolecular hydrogen bonds (e.g., O3–H⋯O5) and intermolecular hydrogen bonds (e.g., O6–H⋯O3′), which collectively contribute to a high Young’s modulus and elevated cohesive energy density [112,113]. The supramolecular organization of cellulose thus provides an intrinsically rigid backbone suitable for structural reinforcement in bio-adhesive matrices. Starch, in contrast, consists of two major fractions: amylose and amylopectin. Amylose is primarily linear and capable of adopting helical conformations, whereas amylopectin exhibits a highly branched structure through α(1 → 6) linkages. The stereochemical distinction between α(1 → 4) and β(1 → 4) linkages significantly influences molecular packing, swelling behavior, and gel formation, all of which directly affect adhesion mechanisms and film-forming performance in starch-based adhesives [83,114]. The semi-crystalline lamellar arrangement of amylopectin further determines viscoelastic properties and interfacial wetting behavior.

The abundance of hydroxyl groups renders carbohydrates highly amenable to chemical modification, including esterification, etherification, and periodate oxidation. Periodate oxidation generates dialdehyde functionalities capable of participating in Schiff base reactions or acetal crosslinking, thereby increasing network density and reducing free volume within the polymer matrix [109,113,114,115]. Such molecular engineering strategies are essential for enhancing water resistance, mechanical strength, and dimensional stability in bio-based adhesive systems.

#### 3.3.2. Interfacial Adhesion Mechanisms of Cellulose and Nanocellulose

Nanocellulose materials, including cellulose nanofibrils (CNF) and cellulose nanocrystals (CNC), possess exceptionally high specific surface areas (>100 m^2^ g^−1^) and large aspect ratios, enabling the formation of percolated hydrogen-bonding networks within adhesive matrices [115,116,117]. These nanostructures enhance stress transfer efficiency and reinforce the polymer network at the nanoscale.

Adhesion at wood–adhesive interfaces is governed by multiple synergistic mechanisms. First, hydrogen bonding occurs between hydroxyl groups of cellulose and phenolic or hydroxyl groups in lignin and hemicellulose [114,117]. Second, mechanical interlocking arises from adhesive penetration into the porous wood microstructure. Third, interdiffusion at the nanoscale can occur when polymer chains exhibit sufficient mobility to entangle across the interface [118]. Collectively, these mechanisms determine the overall interfacial shear strength. The incorporation of nanocellulose enhances stress transfer via a shear-lag mechanism and mitigates crack propagation through crack deflection and energy dissipation processes, thereby significantly improving fracture toughness [119]. From a sustainability perspective, replacing petrochemical resins with cellulose-based reinforcements reduces embodied carbon and fossil resource dependence, contributing to the transition toward low-carbon material systems [23].

#### 3.3.3. Physicochemical Modification and Network Formation in Starch-Based Adhesives

Native starch exhibits high hydrophilicity due to extensive hydrogen bonding between hydroxyl groups and water molecules, leading to plasticization and a reduction in glass transition temperature (T_g_) [120]. This intrinsic moisture sensitivity limits its structural durability under humid conditions.

Chemical crosslinking using dialdehydes or polycarboxylic acids forms covalent bridges between polymer chains through acetal or ester linkages, thereby increasing crosslink density (v_e_) and restricting chain mobility [44,120]. At the thermodynamic level, an increase in crosslink density elevates the Gibbs free energy of mixing (ΔG_mix_) suppressing polymer–water miscibility and enhancing water resistance. Concurrently, cohesive strength increases due to greater entanglement density and secondary intermolecular interactions [121]. Such molecular modifications not only improve mechanical integrity and hydrothermal stability but also reduce reliance on formaldehyde-emitting synthetic binders, which are associated with volatile organic compound (VOC) emissions and adverse health effects [23,44]. Consequently, starch-based adhesive engineering represents a viable pathway for environmentally responsible material innovation across the product life cycle [121,122].

#### 3.3.4. Hemicellulose Reactivity and Functional Blending Strategies

Hemicellulose is an amorphous heteropolysaccharide composed primarily of xylose, mannose, arabinose, galactose, and glucose units linked through branched polymeric structures. Unlike cellulose, hemicellulose possesses a non-crystalline and highly irregular molecular architecture characterized by abundant hydroxyl groups, acetyl substituents, uronic acids, and arabinosyl side chains, all of which contribute to its high chemical reactivity and structural versatility [123,124,125]. The relatively low degree of polymerization and branched configuration of hemicellulose confer greater molecular mobility and chain flexibility compared with cellulose, enabling improved wetting behavior, interfacial contact, and rheological tunability in adhesive formulations [124]. From a polymer science perspective, the abundant hydroxyl functionalities within hemicellulose facilitate extensive intermolecular hydrogen bonding with lignocellulosic substrates, thereby contributing to adhesive cohesion and interfacial adhesion. In addition, reactive side groups such as acetyl and uronic acid moieties enable various chemical modification pathways, including esterification, etherification, oxidation, graft copolymerization, and crosslinking reactions with multifunctional curing agents [123]. These modification strategies are essential for overcoming the inherent hydrophilicity and limited moisture resistance of native hemicellulose-based adhesives.

The amorphous structure of hemicellulose also provides significant advantages in the formation of hybrid polymeric adhesive networks. When blended with proteins, starch, lignin, tannins, or synthetic bio-based crosslinkers, hemicellulose can participate in the formation of semi-interpenetrating polymer networks (semi-IPNs) and highly entangled macromolecular structures. Such hybrid systems improve cohesive energy density, stress transfer efficiency, and network homogeneity without excessively increasing brittleness. Consequently, hemicellulose-containing adhesive systems often exhibit improved flexibility, enhanced crack resistance, and more uniform stress redistribution under mechanical loading conditions [124,125]. Moreover, hemicellulose plays an important role in controlling adhesive rheology and processing behavior. Owing to its flexible chain conformation and tunable molecular weight distribution, hemicellulose can regulate viscosity, flowability, and penetration into porous wood substrates during hot pressing. Proper rheological control is particularly important for achieving optimal adhesive spreading, intimate substrate contact, and uniform bond-line formation in wood composites and engineered panels. However, excessive hydrophilicity and low intrinsic crosslink density may still result in poor wet strength, dimensional instability, and susceptibility to hydrothermal degradation unless suitable chemical modifications or hybridization approaches are employed.

Recent studies have increasingly focused on integrating hemicellulose into multifunctional hybrid adhesive systems to improve both performance and sustainability. Oxidized hemicellulose, aldehyde-functionalized hemicellulose, and hemicellulose-based graft copolymers have demonstrated enhanced crosslinking efficiency and improved compatibility with protein- and lignin-based adhesive matrices. Furthermore, incorporation of nanocellulose, lignin nanoparticles, or polyphenolic compounds into hemicellulose-rich systems has been shown to increase network compactness, reduce water uptake, and enhance thermal stability. From a sustainability perspective, hemicellulose represents a highly attractive renewable feedstock because it is abundantly available in agricultural residues, forestry by-products, and lignocellulosic biomass streams. Large quantities of hemicellulose can be recovered from rice straw, sugarcane bagasse, oil palm residues, corn stover, and hardwood processing wastes, thereby supporting biomass valorization and circular bioeconomy strategies [123,125]. The utilization of hemicellulose in adhesive formulations contributes to improved resource efficiency, reduced dependence on petrochemical polymers, and lower greenhouse gas emissions associated with wood composite manufacturing. Consequently, hemicellulose-based and hemicellulose-modified adhesive systems are increasingly recognized as promising candidates for the development of sustainable, low-carbon, and formaldehyde-free wood adhesives suitable for next-generation engineered wood products.

### 3.4. Tannins

**Figure 6:**
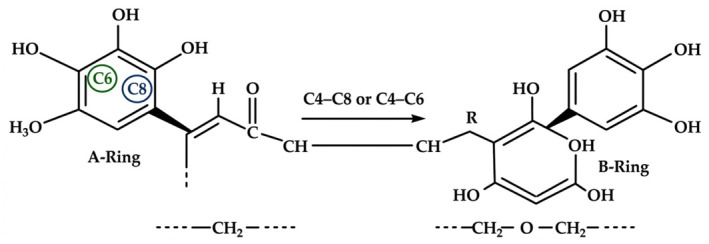
Chemical structure of condensed tannin (proanthocyanidin).

Tannins are naturally occurring plant-derived polyphenols widely recognized as promising renewable precursors for thermosetting wood adhesives. Owing to their high aromatic content and dense distribution of phenolic hydroxyl groups, tannins exhibit chemical reactivity comparable to conventional phenol–formaldehyde (PF) systems [126,127]. This structural similarity enables partial substitution of fossil-derived phenol while maintaining desirable bonding performance. Among naturally occurring tannins, condensed tannins (proanthocyanidins) are particularly suitable for adhesive applications because of their superior thermal stability and high electrophilic substitution reactivity [127]. As illustrated in Figure 6, condensed tannins consist of flavan-3-ol repeating units linked through C4–C8 or C4–C6 interflavan bonds. Each structural unit contains a resorcinol-type A-ring and a catechol- or pyrogallol-type B-ring bearing multiple phenolic hydroxyl groups. These functional groups govern curing behavior and network formation, directly influencing final adhesive performance [127,128].

#### 3.4.1. Molecular Structure and Chemical Reactivity

The molecular architecture shown in Figure 6 explains the high reactivity of condensed tannins. The electron-donating hydroxyl substituents activate the aromatic rings toward electrophilic substitution, particularly at the C6 and C8 positions of the A-ring [21,101]. Under alkaline conditions, these activated sites readily react with formaldehyde to form hydroxymethylated intermediates.

Subsequent condensation reactions generate methylene (–CH_2_–) and methylene ether (–CH_2_–O–CH_2_–) bridges through step-growth polycondensation, progressively forming a three-dimensional thermoset network [4]. Due to the intrinsic multifunctionality of tannin macromolecules, multiple reactive sites are present within each repeating unit, leading to rapid gelation and efficient crosslink formation even at reduced formaldehyde loadings [101,127,129]. However, steric hindrance arising from interflavan linkages and the pre-existing oligomeric backbone may influence diffusion-controlled reaction stages at high conversion levels. As curing proceeds, vitrification may occur earlier than in monomeric phenol systems, thereby affecting final crosslink density and network homogeneity [21,129].

#### 3.4.2. Thermomechanical Performance and Adhesive Properties

The crosslink density developed during curing directly determines the thermomechanical performance of tannin-based adhesives. According to classical polymer network theory [130,131], an increase in crosslink density enhances stiffness and elevates the glass transition temperature (T_g_) due to restricted molecular mobility. Consequently, properly formulated tannin–formaldehyde systems can achieve bonding strengths comparable to PF resins in plywood and particleboard applications [4].

The rigid aromatic backbone of condensed tannins further contributes to improved thermal resistance and dimensional stability. Nevertheless, excessive crosslinking may reduce toughness, necessitating optimization of curing temperature, pH, and aldehyde content to balance stiffness and fracture resistance [4,21,131]. Furthermore, the high density of phenolic hydroxyl groups enhances interfacial interactions with lignocellulosic substrates through hydrogen bonding and potential covalent linkages, thereby improving adhesion performance in engineered wood composites [21].

#### 3.4.3. Environmental and Sustainability Implications

Beyond performance considerations, tannin-based adhesives provide significant environmental advantages. Tannins are extracted from renewable biomass resources such as bark and heartwood, contributing to biogenic carbon storage within the polymer matrix [4]. Partial substitution of fossil-derived phenol reduces dependence on petrochemical feedstocks and lowers embodied carbon in adhesive formulations.

In addition, the high intrinsic reactivity of tannins allows reduced formaldehyde usage, potentially decreasing free formaldehyde emissions from wood-based panels [21,131]. Life cycle assessment (LCA) studies indicate that incorporating bio-based phenolic substitutes can lower global warming potential (GWP) under cradle-to-gate system boundaries [131,132,133,134]. Overall, the structural features presented in Figure 6 underpin not only the curing mechanism and thermomechanical performance of tannin-based adhesives but also their role in advancing sustainable and low-carbon materials engineering strategies [134].

### 3.5. Comparative Structure–Property–Sustainability Relationships of Major Bio-Based Wood Adhesives

Bio-based wood adhesives can generally be classified into four major categories: protein-based adhesives, carbohydrate-based adhesives, lignin-based adhesives, and tannin-based adhesives. Each adhesive system possesses distinct molecular structures, reactive functional groups, curing mechanisms, and interfacial interaction pathways, which directly influence adhesive performance, durability, environmental impact, and industrial applicability.

Protein-based adhesives, particularly those derived from soybean, cottonseed, blood protein, and gelatin, are characterized by abundant amino, carboxyl, hydroxyl, and sulfhydryl functional groups. These functionalities enable strong intermolecular hydrogen bonding and covalent crosslinking interactions with lignocellulosic substrates. During curing, protein unfolding exposes reactive side chains that participate in network formation through condensation, Maillard reactions, epoxy crosslinking, aldehyde reactions, or polycarboxylic acid coupling. Such mechanisms contribute to improved dry bonding strength and interfacial adhesion with cellulose-rich wood surfaces. However, the inherently hydrophilic nature of protein macromolecules often leads to moisture sensitivity and reduced wet strength unless additional crosslinking agents or hydrophobic modifications are introduced. Consequently, current research focuses heavily on enhancing hydrothermal stability through multifunctional crosslinkers, nanostructured reinforcements, and semi-interpenetrating polymer network formation.

Carbohydrate-based adhesives, including starch, cellulose derivatives, hemicellulose, and chitosan systems, primarily rely on hydroxyl-rich polysaccharide structures. Their adhesive behavior is governed by hydrogen bonding, chain entanglement, gelatinization, and chemically induced network formation. Oxidation, esterification, etherification, and graft polymerization are commonly employed to increase crosslink density and reduce hydrophilicity. Starch-based systems exhibit good dry adhesion and relatively low cost, making them attractive for interior-grade applications. Nevertheless, excessive water absorption, dimensional instability, and biological degradation remain important limitations under humid conditions. Cellulose nanomaterials and modified polysaccharides have therefore been increasingly incorporated to improve mechanical reinforcement, rheological stability, and moisture resistance.

Lignin-based adhesives differ substantially from protein and carbohydrate systems because of their highly aromatic and phenolic molecular architecture. The presence of phenolic hydroxyl groups enables lignin to partially substitute petroleum-derived phenol in thermosetting resin systems such as phenol–formaldehyde adhesives. Condensation reactions between activated aromatic sites and aldehydes generate highly crosslinked three-dimensional polymer networks with enhanced thermal stability and water resistance. However, lignin heterogeneity, relatively low reactivity, steric hindrance, and batch-to-batch variability often limit curing efficiency and industrial process consistency. Chemical depolymerization, phenolation, methylolation, and oxidative activation are therefore widely applied to improve lignin reactivity and compatibility with curing systems.

Tannin-based adhesives, particularly condensed tannins derived from bark and plant polyphenols, possess highly reactive flavonoid structures rich in phenolic hydroxyl groups. These structures readily undergo self-condensation and aldehyde-assisted polymerization reactions, enabling rapid curing and strong adhesive network formation. Tannin adhesives generally exhibit excellent dry bonding strength, favorable thermal resistance, and relatively low formaldehyde emissions. Their aromatic nature also contributes to improved hydrophobicity compared with starch- or protein-based systems. However, rapid viscosity increase, limited storage stability, and raw material variability remain technical barriers for large-scale industrial implementation. Hybridization with proteins, lignin, or synthetic crosslinkers has been explored to overcome these limitations and improve processing behavior.

Recent technological developments increasingly emphasize hybrid bio-based adhesive systems that combine complementary molecular functionalities from multiple biomass sources. Protein–carbohydrate hybrid systems, lignin-modified soy adhesives, tannin–protein networks, and semi-interpenetrating polymer structures have demonstrated improved crosslink density, enhanced moisture resistance, reduced formaldehyde emissions, and superior mechanical performance. From a sustainability perspective, these hybrid systems also support reduced fossil carbon dependence, lower volatile organic compound (VOC) emissions, and reduced global warming potential (GWP) relative to conventional petrochemical adhesives (see Table 2 and Table 3). The performance of bio-based wood adhesives is fundamentally governed by factors summarized in Table 4.

Accordingly, future development of bio-based wood adhesives will likely focus on molecular engineering approaches that integrate renewable aromatic structures, multifunctional crosslinking chemistry, nanostructured reinforcement, and predictive structure–property modeling to achieve industrial-scale, high-performance, low-carbon adhesive systems.

### 3.6. Sustainability and Life Cycle Assessment: A Critical Perspective

The transition toward bio-based wood adhesives within the Bio–Circular–Green (BCG) economy framework is frequently driven by the assumption that feedstock renewability directly equates to environmental friendliness. However, a rigorous synthesis of current Life Cycle Assessment (LCA) literature (as systematically compiled in Table 5) reveals sophisticated environmental trade-offs that challenge this binary perspective. When subjected to a standardized functional unit and explicit system boundaries, bio-based formulations often display distinct environmental hotspots that can occasionally negate their bio-derived benefits.

A primary critical concern emerges from the upstream agricultural phase of plant-derived proteins. While isolated soy protein (ISP) adhesives effectively eliminate hazardous formaldehyde emissions during wood panel hot-pressing, their holistic LCA profile reveals that certain plant protein-derived formulations can display global warming potential (GWP) metrics that noticeably surpass the carbon baseline of conventional petrochemical alternatives like urea-formaldehyde (UF) resins, as represented by the specific data ranges detailed in Table 5. This unexpected carbon penalty is directly attributed to major agricultural hotspots, specifically intensive land-use conversion, mechanical farming operations, and the high application rates of synthetic nitrogen-based fertilizers during crop cultivation. Furthermore, because pristine plant biopolymers inherently possess compact, highly folded macromolecular architectures, they suffer from poor moisture resistance in their native states. Overcoming these performance and structural bottlenecks requires aggressive chemical denaturing and downstream refinement, processes that consume significant industrial electricity and thermal energy, thereby shifting the environmental burden from the formulation phase back to the processing phase.

Another critical bottleneck lies within the crosslinking chemistry mandated by the polymer network. Due to the inherently low moisture stability of raw condensed tannins and lignins, the incorporation of synthetic, fossil-derived crosslinkers or network modifiers remains functionally mandatory to meet structural standards for engineered wood panels. The environmental impacts of these systems, represented by the data ranges in Table 5, are heavily dictated by the synthesis and integration of these hardeners, which introduce synthetic toxicological and carbon-heavy components into an otherwise natural adhesive network.

Conversely, the use of industrial by-products, such as Kraft or organosolv lignin, offers a more viable circular pathway. When utilized to partially substitute petro-based phenol in PF resins, the total environmental footprint can be noticeably reduced. This net advantage is structurally possible because technical lignin is treated as an industrial waste product from the paper pulping sector, thus escaping the heavy carbon and land-use allocations associated with primary agricultural cultivation while supporting the circular economy model within wood-based panel lines.

In conclusion, the empirical ranges maintained in Table 5 underscore that the true eco-efficiency of next-generation wood adhesives cannot be evaluated solely on the basis of bio-content. Future research must urgently close the gap between structural performance and environmental impact by validating non-toxic, bio-derived crosslinkers and shifting manufacturing processes toward renewable energy grids. Without these holistic interventions, the complete substitution of fossil resources remains an incomplete solution to achieving true net-zero targets.

## 4. Properties of Structural Wood Adhesives

The development of bio-based adhesives for structural wood applications requires a rigorous understanding of polymer science, interfacial mechanics, and curing network formation. For load-bearing applications, the adhesive must exhibit mechanical performance comparable to or exceeding that of the wood substrate, such that failure preferentially occurs within the wood rather than at the bond-line [4]. Shear strength, creep resistance, and long-term viscoelastic stability are therefore critical indicators of structural performance.

From a polymer science perspective, adhesive performance is governed by molecular architecture, crosslink density, and curing kinetics. The rheological properties of the adhesive determine its penetration into the wood lumen and cell wall microstructure, thereby influencing mechanical interlocking and effective stress transfer across the interface [78,88]. Insufficient penetration limits interfacial adhesion, whereas excessive penetration may result in a starved bondline and reduced cohesive strength. Recent studies in Polymers have demonstrated that tailoring functional group density and crosslinking pathways in bio-based thermosets significantly enhances shear strength and hydrolytic stability [118,126,127,128]. Moisture resistance remains a decisive parameter in structural wood bonding, as wood undergoes anisotropic dimensional changes in response to humidity fluctuations. These dimensional variations generate internal stresses at the adhesive interface, potentially leading to delamination. Consequently, structural adhesives must exhibit low water affinity, high hydrolytic stability, and sufficient viscoelastic relaxation capacity to accommodate stress without compromising network integrity [4,133]. Chemical modification of polysaccharides—such as cellulose and hemicellulose—has been shown to reduce hygroscopicity and improve dimensional stability [129,131]. Given that carbohydrates constitute the most abundant class of natural polymers in biomass [14,73]; their functionalization provides a promising pathway for high-performance bio-adhesive systems. In addition to mechanical durability, modern adhesives must comply with increasingly stringent environmental and health regulations by minimizing volatile organic compound (VOC) emissions and free formaldehyde releases [83,141]. Life Cycle Assessment (LCA) studies have revealed that substituting fossil-derived resins with bio-based systems can significantly reduce global warming potential (GWP), particularly when high biogenic carbon content is retained within the cured network [21,139]. These findings position bio-based structural adhesives as a key material innovation in climate-conscious construction.

The performance of bio-based wood adhesives is strongly governed by polymer architecture, reactive functional group density, curing chemistry, and interfacial interactions with lignocellulosic substrates. Compared with conventional petroleum-based adhesive systems such as urea–formaldehyde (UF), melamine–urea–formaldehyde (MUF), phenol–formaldehyde (PF), and polymeric methylene diphenyl diisocyanate (pMDI), bio-based adhesives generally exhibit lower hydrothermal durability and slower curing behavior; however, they provide substantial environmental advantages, including lower volatile organic compound (VOC) emissions, reduced fossil carbon dependence, and improved sustainability profiles.

Protein-based adhesives, particularly soy protein and cottonseed protein systems, exhibit moderate-to-high dry shear strength due to extensive hydrogen bonding, electrostatic interactions, and covalent crosslinking with cellulose and lignin components of wood substrates. Nevertheless, their wet shear strength and delamination resistance remain lower than those of PF and pMDI systems because of the intrinsic hydrophilicity of peptide and amino acid functional groups. Chemical modification using epoxies, aldehydes, polycarboxylic acids, tannins, or lignin-derived crosslinkers substantially improves crosslink density, reduces water uptake, and enhances wet adhesion performance.

Carbohydrate-based adhesives, including starch and cellulose derivatives, generally display relatively high viscosity and short-to-moderate pot life because of hydroxyl-rich polysaccharide structures and rapid hydrogen-bond network formation. Their dry bonding strength can approach UF-type systems after esterification or oxidation treatments; however, moisture resistance and long-term dimensional stability remain key limitations unless multifunctional crosslinking strategies are employed.

Lignin- and tannin-based adhesives exhibit superior thermal stability and lower VOC emissions because of their aromatic phenolic structures. Condensed tannins and modified lignin can partially replace phenol in thermosetting resin systems and provide improved water resistance compared with protein- and starch-based adhesives. However, curing kinetics are often slower and may require elevated curing temperatures or catalyst-assisted condensation reactions. Hybrid adhesive systems integrating proteins, carbohydrates, lignin, and tannins increasingly demonstrate balanced dry/wet performance, reduced formaldehyde emission, and improved industrial applicability.

In contrast, conventional UF adhesives exhibit rapid curing, low viscosity, and low manufacturing cost but suffer from poor moisture resistance and relatively high formaldehyde emissions. MUF systems improve hydrothermal durability compared with UF through melamine incorporation, whereas PF adhesives provide excellent weather resistance and thermal stability at the expense of darker coloration and higher curing temperatures. pMDI systems generally exhibit the highest bonding performance and moisture resistance because of highly reactive isocyanate chemistry, although concerns remain regarding occupational exposure, petrochemical origin, and processing safety. The overall performance trends of bio-based wood adhesives are summarized in Table 6. Additionally, Table 7 presents a performance comparison between bio-based adhesives and conventional industrial adhesive systems.

## 5. Potential of Bio-Based Adhesives Within the Bio–Circular–Green (BCG) Economy Framework

The advancement of bio-based adhesives under the Bio–Circular–Green (BCG) economy paradigm extends beyond fossil substitution. It encompasses molecular-level design, resource valorization, and quantitative carbon accounting across the product life cycle. Within this framework, polymer chemistry and sustainability metrics converge to optimize both performance and environmental impact.

Plant-derived proteins, particularly soy protein, represent one of the most industrially viable bio-based adhesive precursors. Their abundant reactive functional groups enable chemical crosslinking and structural reinforcement through co-reactants or network modifiers, thereby enhancing water resistance and mechanical strength [36,106]. Recent research published in Industrial Crops and Products and Polymers indicates that optimized soy-protein-based thermosets can achieve structural bonding performance comparable to conventional urea–formaldehyde systems under controlled curing conditions [22,30,31,32,33,34,35,84]. Lignin, an aromatic biopolymer derived from lignocellulosic biomass, offers substantial potential as a partial phenol substitute in phenol–formaldehyde resins. However, its relatively low reactivity necessitates chemical modification, such as hydroxyl-methylation or phenolation, to improve compatibility and crosslinking efficiency [22,142]. Recent contributions in Green Chemistry have emphasized lignin valorization strategies that enhance reactivity while reducing the carbon footprint associated with petrochemical phenol production [32,33,34,142]. Such approaches exemplify circular economy principles by converting industrial by-products into high-value functional materials. Tannins, naturally occurring polyphenols, are another promising bio-aromatic platform for thermosetting adhesive systems. Their high phenolic hydroxyl content enables efficient network formation without reliance on free formaldehyde [36,84]. Advances in tannin-based adhesive formulations reported in Industrial Crops and Products have demonstrated improved mechanical stability and moisture resistance suitable for structural applications [84,89]. From a Net-Zero perspective, increasing the proportion of biogenic carbon in adhesive systems contributes to reducing embodied carbon in engineered wood products. Quantitative carbon analysis through LCA provides a robust methodology to evaluate cumulative greenhouse gas emissions, carbon storage duration, and end-of-life scenarios [14,132,133,134]. Integrating high-performance polymer design with carbon-efficient material sourcing aligns adhesive innovation with broader sustainability targets, including responsible production and climate action goals. In summary, the future of structural wood adhesives lies in the integration of advanced polymer chemistry, process engineering, and life-cycle-based carbon assessment. Such a multidisciplinary strategy enables the development of high-performance, low-emission adhesive systems that support the transition toward a low-carbon, circular bio-economy.

## 6. Conclusions

Bio-based wood adhesives derived from proteins, carbohydrates, lignin, and tannins exhibit significant potential as partial or full substitutes for petrochemical resins. Adhesive performance is governed by reactive functional group availability, crosslink density, and network homogeneity. Chemical modification strategies—including oxidation, esterification, phenolic substitution, and multifunctional crosslinking—are critical for enhancing moisture resistance and mechanical strength. Lignin and tannins provide bio-aromatic structures suitable for thermosetting systems, whereas protein and carbohydrate matrices require controlled crosslinking to mitigate hydrophilicity. Hybrid and semi-interpenetrating polymer networks offer a balanced approach to achieving high bonding strength, dimensional stability, and reduced formaldehyde emission. Future research should prioritize quantitative correlations between molecular structure, curing kinetics, crosslink density, and durability metrics, coupled with life cycle assessment parameters. Such integration will enable predictive optimization of polymer architecture and accelerate industrial deployment of high-performance, low-carbon bio-based adhesive systems.

## Figures and Tables

**Table 1:** Comparative summary of plant protein-based wood adhesives.

Protein Sources	Origin/ Availability	Modification Strategy	Key Performance Improvements	Sustainability Contribution
Soybean protein	Major agricultural crop; abundant	Chemical/enzymatic modification; epoxy, MF, pMDI cross-linking	Improved water resistance, thermal stability, and bonding strength	Reduces petrochemical resin use; supports SDGs 3, 12
Cottonseed protein	Non-food agricultural by-product	Acid treatment; small-molecule additives	Enhanced dry and wet shear strength	Valorization of toxic by-products; circular bio-economy
Camelina protein	Oilseed processing residue	PAE cross-linking	Increased wet strength and reduced protein solubility	Waste utilization; low-carbon material pathway
Pea protein	Food-grade crop; renewable	Additives and cross-linkers	Comparable dry/wet strength to soy protein	Renewable alternative protein source
Jatropha protein	Biodiesel industry by-product	Alkaline treatment; polymer blending	Improved viscosity, penetration, and shear strength	Bioenergy residue valorization
Canola protein	Oilseed crop residue	Surfactants; graft polymerization	Enhanced adhesion, rheology, and water resistance	Circular use of oilseed biomass
Zein (corn)	Corn processing by-product	Dispersants and cross-linkers	Improved cohesion and moisture tolerance	Food industry waste valorization
Wheat gluten	Starch industry by-product	Cross-linking and plasticization	Increased flexibility and reduced swelling	Sustainable agro-industrial material

Note: melamine-formaldehyde (MF); polymeric methylene diphenyl diisocyanate (pMDI); polyamidoamine–epichlorohydrin (PAE); sustainable development goals (SDGs).

**Table 2:** Comparative analysis of major bio-based wood adhesives based on characteristics, crosslinking mechanisms, curing behavior, and interfacial adhesion.

Adhesive Type	Chemical Characteristics	Bonding/Crosslinking Mechanisms	Curing Behavior	Interfacial Adhesion
Protein-based adhesives (soy, cottonseed, blood protein, gelatin)	Amino, carboxyl, hydroxyl, sulfhydryl groups; polypeptide structure	Hydrogen bonding, covalent crosslinking, Maillard reactions, epoxy or aldehyde coupling	Heat-induced unfolding and network formation	Strong interaction with cellulose and lignin via polar groups
Carbohydrate-based adhesives (starch, cellulose, hemicellulose, chitosan)	Hydroxyl-rich polysaccharide chains	Hydrogen bonding, gelatinization, esterification, etherification, graft polymerization	Thermal gelatinization and chemical curing	Good surface wetting due to hydrophilicity
Lignin-based adhesives	Aromatic phenolic polymer with methoxy and hydroxyl groups	Phenolic condensation, aldehyde crosslinking, thermosetting network formation	Slower curing due to steric hindrance and low reactivity	Strong aromatic interactions with lignocellulosic surfaces
Tannin-based adhesives	Polyphenolic flavonoid structures with high phenolic hydroxyl density	Self-condensation, aldehyde-assisted polymerization	Rapid curing and high crosslink density	Strong adhesion via aromatic and hydrogen bonding interactions
Hybrid bio-based adhesives (protein–carbohydrate, lignin–modified, tannin hybrids)	Multiple complementary reactive functionalities	Interpenetrating polymer networks, synergistic covalent and hydrogen bonding	Tailorable curing kinetics and network topology	Enhanced interfacial compatibility and stress transfer

**Table 3:** Comparative analysis of major bio-based wood adhesives based on bond strength, durability, environmental profiles, and industrial readiness.

Adhesive Type	Dry Bond Strength	Wet Bond Strength	Durability Limitations	Environmental Profile	Industrial Readiness
Protein-based adhesives (soy, cottonseed, blood protein, gelatin)	High	Moderate to low unless modified	Moisture sensitivity, hydrothermal instability, biodegradation	Renewable, low VOC, low fossil carbon content	Medium–High
Carbohydrate-based adhesives (starch, cellulose, hemicellulose, chitosan)	Moderate–High	Moderate	Water absorption, dimensional instability, microbial susceptibility	Highly renewable and biodegradable	Medium
Lignin-based adhesives	High	High	Heterogeneity, limited reactivity, curing inconsistency	Reduced fossil phenol demand and lower carbon footprint	Medium–High
Tannin-based adhesives	High	High	High viscosity, storage instability, raw material variability	Low formaldehyde emission, renewable aromatic resource	High
Hybrid bio-based adhesives (protein–carbohydrate, lignin–modified, tannin hybrids)	Very high	High	Processing complexity and formulation optimization challenges	Lowest GWP and VOC potential among bio-based systems	Emerging–High

**Table 4:** Overview of structure–property–sustainability relationships in bio-based wood adhesives.

Governing Factor	Key Characteristics/ Mechanisms	Impact on Adhesive Performance
Reactive functional group density	High concentration of amino, hydroxyl, and phenolic groups	– Improves interfacial adhesion – Enhances crosslinking capability
Crosslink density and network topology	Dense three-dimensional polymer networks	– Enhances water resistance – Improves thermal stability – Increases mechanical durability
Hydrophilic–hydrophobic balance	Optimization of moisture-sensitive and moisture-resistant elements	– Prevents reduction of wet strength and dimensional stability (from excessive hydrophilicity) – Aromatic and hydrophobic structures improve moisture resistance
Polymer compatibility and interfacial interactions	Synergistic effects in hybrid systems	– Improves stress distribution – Enhances adhesive penetration into porous wood structures
Sustainability metrics	High bio-based carbon content with lower hazardous emissions	– Reduces formaldehyde usage and VOC emissions – Improves environmental performance – Supports net-zero manufacturing strategies

**Table 5:** The environmental footprint of bio-based systems.

Adhesive System	System Boundary	GWP (kg CO_2_-eq/kg)	Key Sustainability Drivers	Reference
Urea-Formaldehyde (UF)	Cradle-to-Gate	1.10–1.80	Fossil fuel extraction, high energy for formaldehyde synthesis.	[135]
Phenol-Formaldehyde (PF)	Cradle-to-Gate	2.10–2.65	Intensive aromatic processing, significant environmental toxicity.	[136]
Soy Protein (Isolated)	Cradle-to-Gate	1.95–2.40	High energy in protein extraction; high agricultural inputs (fertilizer).	[137]
Lignin-modified PF	Cradle-to-Gate	1.35–1.60	Substitution of phenol reduces GWP but depends on lignin purity.	[138]
Bio-Polyurethane (Vegetable oil)	Cradle-to-Gate	0.85–1.20	Biogenic carbon sequestration; lower process energy.	[139]
Tannin-based (Mimosa)	Cradle-to-Gate	1.45–1.70	Extraction energy and chemical modifiers (e.g., hexamine).	[140]

Note: GWP values are approximated based on ‘Cradle-to-Gate’ boundaries as reported in the respective literature. Variations may occur due to differences in functional units, extraction methods, and energy mix assumptions in each study.

**Table 6:** Overview of structure–performance trends relationships in bio-based wood adhesives.

Performance Factors	Structure–Performance Trends
Dry shear strength	- PF and pMDI remain the highest-performing industrial systems. - Hybrid bio-based systems increasingly approach MUF-level dry bonding performance.
Wet shear strength and delamination resistance	- Strongly correlated with crosslink density and hydrophobic network formation. - Tannin- and lignin-modified systems exhibit the best hydrothermal resistance among bio-based adhesives.
Viscosity and processability	- Polysaccharide-rich adhesives generally possess higher viscosity because of extensive intermolecular hydrogen bonding. - UF and pMDI systems remain advantageous for high-speed industrial processing.
Thermal stability	- Aromatic phenolic structures in lignin, tannins, and PF systems contribute to superior thermal resistance. - Protein and starch systems require chemical reinforcement for elevated-temperature applications.
VOC emissions and sustainability	- Bio-based systems generally exhibit substantially lower VOC emissions and reduced fossil carbon footprint. - Formaldehyde-free hybrid systems demonstrate strong alignment with low-carbon manufacturing and circular bioeconomy frameworks.
Industrial readiness	- UF, MUF, PF, and pMDI remain dominant because of processing reliability and established infrastructure. - Bio-based adhesives are rapidly progressing from laboratory-scale formulations toward pilot-scale and commercial implementation, particularly for interior-grade and low-emission wood composites.

**Table 7:** Comparison of bio-based adhesives and conventional industrial adhesive systems.

Adhesive System	Chemical Functionality	Dry Shear Strength	Wet Shear Strength	Delamination Resistance	Wood Failure	Viscosity	Pot Life	Curing Temperature	Pressing Time	Thermal Stability	VOC Emission	Major Advantages	Major Limitations	Industrial Readiness
		(MPa)	(MPa)		(%)			(°C)	(min)					
Soy Protein Adhesive	Amino, carboxyl, hydroxyl groups	1.5–3.5	0.5–1.8	Moderate	40–85	High	Moderate	110–160	4–10	Moderate	Very low	Renewable, formaldehyde-free, strong dry adhesion	Moisture sensitivity, microbial susceptibility	Pilot to commercial
Cottonseed Protein Adhesive	Protein functional groups	1.2–3.0	0.4–1.5	Moderate	35–80	Moderate–high	Moderate	120–170	5–12	Moderate	Very low	Agricultural by-product utilization	Limited wet durability	Pilot scale
Blood/Gelatin Protein Adhesive	Peptide and amide groups	1.5–3.2	0.5–1.4	Moderate	40–75	Moderate	Short	110–150	5–10	Moderate	Very low	Strong interfacial interactions	Thermal instability, biodegradation	Limited
Starch-Based Adhesive	Hydroxyl-rich polysaccharides	1.0–3.0	0.2–1.2	Low–moderate	30–70	High	Short	100–150	3–8	Low–moderate	Very low	Low cost, biodegradable	High hydrophilicity	Commercial (interior grade)
Cellulose/Hemicellulose Adhesive	Hydroxyl and ether groups	1.2–2.8	0.3–1.0	Low–moderate	30–65	High	Moderate	120–180	5–12	Moderate	Very low	Abundant biomass source	Slow curing, moisture sensitivity	Emerging
Lignin-Based Adhesive	Phenolic aromatic structures	2.0–4.5	1.0–2.5	Good	60–90	Moderate	Long	140–200	5–15	High	Low	High thermal stability, phenol substitution	Lower reactivity than phenol	Commercial blending
Tannin-Based Adhesive	Polyphenolic flavonoid structures	2.5–5.0	1.5–3.0	Good–excellent	70–95	Moderate	Moderate	120–180	4–10	High	Low	Fast condensation, low formaldehyde	Limited raw material consistency	Commercial niche
Hybrid Protein–Carbohydrate Adhesive	Hydrogen bonding + covalent crosslinking	2.5–4.5	1.2–2.8	Good	65–95	Moderate–high	Moderate	120–180	4–10	Moderate–high	Very low	Improved network density and wet strength	Complex formulation optimization	Emerging commercial
Hybrid Lignin–Protein Adhesive	Phenolic–protein crosslinked network	2.8–5.0	1.5–3.2	Good–excellent	70–95	Moderate	Moderate	130–190	5–12	High	Low	Enhanced water resistance and strength	Variable lignin chemistry	Emerging
UF (Urea–Formaldehyde)	Amino thermoset resin	3.0–5.5	0.5–1.5	Poor–moderate	70–100	Low	Long	100–130	2–5	Moderate	High	Low cost, rapid curing	Formaldehyde emission, low moisture durability	Fully commercial
MUF (Melamine–Urea–Formaldehyde)	Amino thermoset with melamine	3.5–6.0	1.5–3.5	Good	80–100	Low–moderate	Moderate	110–150	3–6	High	Moderate	Improved moisture resistance	Still formaldehyde-based	Fully commercial
PF (Phenol–Formaldehyde)	Phenolic thermoset resin	4.0–6.5	3.0–5.0	Excellent	90–100	Moderate	Long	140–180	5–15	Very high	Moderate	Excellent durability and weather resistance	Dark color, higher energy consumption	Fully commercial
pMDI	Isocyanate functionality	5.0–8.0	4.0–7.0	Excellent	90–100	Low	Moderate	80–140	2–6	Very high	Very low	Highest bonding performance	Toxicity and handling concerns	Fully commercial

## Data Availability

No new data were created or analyzed in this study. Data sharing is not applicable to this article.

## Supplementary Materials

The following supporting information can be downloaded at: https://www.mdpi.com/article/10.3390/polym18141689/s1, Table S1. Prisma Cheklist.
