# Supplementary material for: Bio-Based Wood Adhesives: Current Advances in Polymer Architecture and Structure–Property–Sustainability Integration
Source: Polymers (Basel). 2026 Jul 9;18(14):1689. doi: 10.3390/polym18141689 (PMC13419046; doi:10.3390/polym18141689)
Supplement: Supplementary file 1 [file polymers-18-01689-s001.zip › polymers-4304605-supplementary.pdf]

PRISMA 2020 Checklist

Section and Topic	Item #	Table S1. Prisma Cheklist<br>Checklist item	Location where item is reported
**TITLE**			
Title	1	Identify the report as a systematic review.	Reported in the title: p. 1, line 1.
**ABSTRACT**			
Abstract	2	See the PRISMA 2020 for Abstracts checklist.	Reported in the Abstract: p. 1, lines 13–34.
**INTRODUCTION**			
Rationale	3	Describe the rationale for the review in the context of existing knowledge.	Reported in the Introduction: p. 3, lines 102–137.
Objectives	4	Provide an explicit statement of the objective(s) or question(s) the review addresses.	Reported in the Introduction: p. 3, lines 138–141; p. 4, lines 146–148; p. 5, lines 149–156.
**METHODS**			
Eligibility criteria	5	Specify the inclusion and exclusion criteria for the review and how studies were grouped for the syntheses.	Reported in the Introduction: p. 4, Figure 1.
Information sources	6	Specify all databases, registers, websites, organisations, reference lists and other sources searched or consulted to identify studies. Specify the date when each source was last searched or consulted.	Reported in the Introduction: p. 3, lines 102–117; p. 4, Figure 1.
Search strategy	7	Present the full search strategies for all databases, registers and websites, including any filters and limits used.	Reported in the Introduction: p. 3, lines 117–137; p. 4, Figure 1.
Selection process	8	Specify the methods used to decide whether a study met the inclusion criteria of the review, including how many reviewers screened each record and each report retrieved, whether they worked independently, and if applicable, details of automation tools used in the process.	Reported in the Introduction: p. 3, lines 125–137; p. 4, Figure 1.
Data collection process	9	Specify the methods used to collect data from reports, including how many reviewers collected data from each report, whether they worked independently, any processes for obtaining or confirming data from study investigators, and if applicable, details of automation tools used in the process.	Reported in the Introduction: p. 3, lines 125–137.
Data items	10a	List and define all outcomes for which data were sought. Specify whether all results that were compatible with each outcome domain in each study were sought (e.g. for all measures, time points, analyses), and if not, the methods used to decide which results to collect.	Reported in the Introduction: p. 3, lines 102–137; p. 4, Figure 1.
	10b	List and define all other variables for which data were sought (e.g. participant and intervention characteristics, funding sources). Describe any assumptions made about any missing or unclear information.	Reported in the Introduction: p. 4, Figure 1.
Study risk of bias assessment	11	Specify the methods used to assess risk of bias in the included studies, including details of the tool(s) used, how many reviewers assessed each study and whether they worked independently, and if applicable, details of automation tools used in the process.	A preliminary and simplified risk of bias assessment was conducted for all included studies using a core methodological checklist. Each study was evaluated independently by five reviewers. Any disagreements in the assessment scores were resolved through consensus or discussion among the team.
Effect measures	12	Specify for each outcome the effect measure(s) (e.g. risk ratio, mean difference) used in the synthesis or presentation of	Reported in the Introduction: p. 4,

		Table S1. Prisma Cheklist	
**Section and Topic**	**Item #**	**Checklist item**	**Location where item is reported**
		results.	Figure 1.
Synthesis methods	13a	Describe the processes used to decide which studies were eligible for each synthesis (e.g. tabulating the study intervention characteristics and comparing against the planned groups for each synthesis (item #5)).	Reported in the Introduction: p. 3, lines 102–137; p. 4, Figure 1.
	13b	Describe any methods required to prepare the data for presentation or synthesis, such as handling of missing summary statistics, or data conversions.	No data conversions or algebraic manipulations were required, as all included studies reported outcomes using standardized and consistent summary statistics.
	13c	Describe any methods used to tabulate or visually display results of individual studies and syntheses.	The results and characteristics of individual studies were tabulated using standardized tables, as shown on p. 9, line 343; p. 21, line 877; p. 22, line 879; p. 22, line 880 and p. 24, line 934.
	13d	Describe any methods used to synthesize results and provide a rationale for the choice(s). If meta-analysis was performed, describe the model(s), method(s) to identify the presence and extent of statistical heterogeneity, and software package(s) used.	Reported in the Introduction: p. 3, lines 102–137; p. 4, Figure 1.
	13e	Describe any methods used to explore possible causes of heterogeneity among study results (e.g. subgroup analysis, meta-regression).	Reported in the Methodology: p. 5, lines 170–178.
	13f	Describe any sensitivity analyses conducted to assess robustness of the synthesized results.	No formal sensitivity analysis was conducted because the synthesis was primarily qualitative.
Reporting bias assessment	14	Describe any methods used to assess risk of bias due to missing results in a synthesis (arising from reporting biases).	An assessment of reporting bias due to missing results was not conducted because this review focuses on qualitative and descriptive synthesis.
Certainty assessment	15	Describe any methods used to assess certainty (or confidence) in the body of evidence for an outcome.	Reported in the Introduction: p. 4, Figure 1 and in the Methodology: p. 5, lines 158–168.
**RESULTS**			
Study selection	16a	Describe the results of the search and selection process, from the number of records identified in the search to the number of studies included in the review, ideally using a flow diagram.	Reported in the Introduction: p. 3, lines 102–137; p. 4, Figure 1.
	16b	Cite studies that might appear to meet the inclusion criteria, but which were excluded, and explain why they were excluded.	Reported in the Introduction: p. 3, lines 102–137; p. 4, Figure 1.
Study characteristics	17	Cite each included study and present its characteristics.	Reported in the Introduction: p. 3, lines 102–137; p. 4, Figure 1.
Risk of bias in studies	18	Present assessments of risk of bias for each included study.	Not applicable (N/A)
Results of	19	For all outcomes, present, for each study: (a) summary statistics for each group (where appropriate) and (b) an effect	For studies with missing summary

		Table S1. Prisma Cheklist	
**Section and Topic**	**Item #**	**Checklist item**	**Location where item is reported**
individual studies		estimate and its precision (e.g. confidence/credible interval), ideally using structured tables or plots.	statistics, their findings were instead synthesized and presented narratively in the text and tables, as shown on p. 9, line 343; p. 21, line 877; p. 22, line 879; p. 22, line 880 and p. 24, line 934.
Results of syntheses	20a	For each synthesis, briefly summarise the characteristics and risk of bias among contributing studies.	Not applicable (N/A)
	20b	Present results of all statistical syntheses conducted. If meta-analysis was done, present for each the summary estimate and its precision (e.g. confidence/credible interval) and measures of statistical heterogeneity. If comparing groups, describe the direction of the effect.	Reported in the Conclusions: p. 27, lines 1058–1072.
	20c	Present results of all investigations of possible causes of heterogeneity among study results.	Not applicable (N/A)
	20d	Present results of all sensitivity analyses conducted to assess the robustness of the synthesized results.	Reported in the Conclusions: p. 27, lines 1058–1072.
Reporting biases	21	Present assessments of risk of bias due to missing results (arising from reporting biases) for each synthesis assessed.	Not applicable (N/A)
Certainty of evidence	22	Present assessments of certainty (or confidence) in the body of evidence for each outcome assessed.	Reported in the Conclusions: p. 27, lines 1058–1072.
**DISCUSSION**			
Discussion	23a	Provide a general interpretation of the results in the context of other evidence.	Not applicable (N/A)
	23b	Discuss any limitations of the evidence included in the review.	Reported in the Properties of Structural Wood Adhesives: p. 25, lines 981–985.
	23c	Discuss any limitations of the review processes used.	Reported in the Bio-Based Adhesives Derived from Natural Polymers: p. 7, lines 256–260.
	23d	Discuss implications of the results for practice, policy, and future research.	Reported in the Potential of Bio-Based Adhesives within the Bio–Circular–Green (BCG) Economy Framework: p. 27, lines 1053–1056 and in the Conclusions: p. 27, lines 1067–1072.
**OTHER INFORMATION**			
Registration and protocol	24a	Provide registration information for the review, including register name and registration number, or state that the review was not registered.	This review was not registered in any prospective database, and a formal protocol was not publicly published online.
	24b	Indicate where the review protocol can be accessed, or state that a protocol was not prepared.	A formal review protocol was not prepared for this review.
	24c	Describe and explain any amendments to information provided at registration or in the protocol.	Not applicable.

PRISMA 2020 Checklist

Section and Topic	Item #	Checklist item	Location where item is reported
		**Table S1. Prisma Cheklist**	
Support	25	Describe sources of financial or non-financial support for the review, and the role of the funders or sponsors in the review.	This review was funded by King Mongkut's Institute of Technology Ladkrabang (KMITL). Role of the Funder: The funder had no role in study design, data collection and analysis, decision to publish, or preparation of the manuscript.
Competing interests	26	Declare any competing interests of review authors.	Competing interests: All authors declare that they have no competing interests, as shown on p. 30, line 1102.
Availability of data, code and other materials	27	Report which of the following are publicly available and where they can be found: template data collection forms; data extracted from included studies; data used for all analyses; analytic code; any other materials used in the review.	Available upon request (see p. 1, line 12).

From: Page MJ, McKenzie JE, Bossuyt PM, Boutron I, Hoffmann TC, Mulrow CD, et al. The PRISMA 2020 statement: an updated guideline for reporting systematic reviews. BMJ 2021;372:n71. doi: 10.1136/bmj.n71. This work is licensed under CC BY 4.0. To view a copy of this license, visit https://creativecommons.org/licenses/by/4.0/
